# The influence and acting pattern of China's national carbon emission trading scheme on regional ecologicalization efficiency of industry

**DOI:** 10.1038/s41598-022-16185-4

**Published:** 2022-07-13

**Authors:** Chun Fu, Yuerong Huang, Yiwei Zheng, Chuanyong Luo

**Affiliations:** 1 School of Public Policy and Management, Nanchang University, Nanchang, 330000 China; 2 School of Foreign Studies, Nanchang University, Nanchang, 330000 China

**Keywords:** Ecology, Environmental social sciences

## Abstract

To achieve the goal of energy conservation and emission reduction, China has launched its national carbon-emission trading scheme (ETS). Therefore, it is of great significance to evaluate the implementation of ETS from the perspective of regional ecologicalization efficiency of industry. Based on the panel data of 30 provinces and cities from 2007 to 2019, this study takes the SBM-DEA model to measure the regional ecologicalization efficiency of industry, uses the Difference-in-Difference (DID) model to evaluate the influence of ETS on the regional ecologicalization efficiency of industry, and verifies the outcome by propensity score matching method and counterfactual test. The mediating effect model is used to test the acting pattern of ETS on the regional ecologicalization efficiency of industry. The results show that: (1) ETS can significantly improve the ecologicalization efficiency of industry and shows regional heterogeneity. (2) ETS promotes regional energy conservation, emission reduction, and economic development because of the upgrading of structural optimization, agglomeration of resource synergy, and optimization of ecology. This study offers penetrating insights into the positive operation of the carbon market and provides some useful hints for the development of China’s regional economy.

## Introduction

To deal with the greenhouse effect, a global environmental problem caused by human manufacturing and life, governments around the world consciously participate in global ecological management. At present, governments that commit to “go net zero” plan to reduce about 90% of global greenhouse gas emissions^1^. Resulting in the most carbon emissions worldwide, China has promised to strive for making carbon dioxide emissions reach a peak before 2030 and reach net-zero in 2060^2^. Bill Gates thinks that the key to reaching net-zero is to reduce the green premium which means a cap on the amount companies are willing to pay for carbon emissions. Green premium and carbon price are closely related: since some enterprises will choose not to reduce emission due to high emission reduction costs, the carbon trading market can use carbon price to intervene. Taking carbon trading as the carrier can help to reduce the green premium and make policy have its effect^3^. Since the global net human-caused emissions of carbon dioxide (CO2) need to reach net-zero around 2050, carbon-emission trading scheme (ETS) are becoming a common choice for policymakers. By the end of 2021, 25 countries’ ETS have covered 17% of global greenhouse gas emissions, among which the European ETS is the most mature and largest^4^. What's more, more and more developing countries are making contributions to the goal of net zero. The government, market and society are compared to the sustainable hands: the government can integrate social resources; the market mechanism is conducive to improving the utilization efficiency of resources and environment; the society can promote the concept of sustainable development. They work in harmony and finally realize the sustainable development of industrial economy. For example, China's national ETS came into operation in 2021 and the carbon cap-and-trade system was put into practice in July. Besides, ETS is on the agenda in some low-income countries such as Vietnam. To achieve the emission reduction target and keep sustainable economic growth, the Chinese government has been committed to exploring emission reduction actions. In 2011, the National Development and Reform Commission issued the *Program for the establishment of a national carbon emissions trading market*, deciding to build regional pilot markets in seven provinces and cities including Beijing, Shanghai, and Shenzhen, and use market mechanisms to meet emission reduction targets. Since 2013, China has carried out ETS in seven regions, including Beijing, Shanghai, Tianjin, Chongqing, Hubei, and Guangdong.

In the regions and industries covered by ETS, the clean production adopted by enterprises affects the ecological level of the whole industry, and in turn, the ecologicalization of the industry also proves the effectiveness of ETS. Research shows that the main factors affecting enterprises’ clean production are carbon price and decarbonization technology. Under the carbon cap-and-trade system, enterprises tend to produce products with high carbon efficiency, so as to make more profits by selling or limiting the purchase of carbon permits^5^. In addition, enterprises that could adopt clean production under ETS need to meet certain objective conditions such as decarbonization technology. Unfortunately, the current research is insufficient to show that ETS could promote decarbonization which may ultimately affect the win–win situation of economic growth and ecological optimization^6,7^.

With the rapid development of China's industrialization process, it is necessary to integrate industrial ecology into industrial development. Industrial system and natural environment interact with each other, prompting elements in the industrial system to change and evolve, and forming a comprehensive, stable and coordinated industrial innovation ecosystem. Industrial ecology not only emphasizes the internal mechanisms, but also the external effects of people or policy. To realize the economic growth, resource consumption reduction, and net-zero, various regional industries need to break the traditional mode, excise management in an innovative way, and strengthen internal communication, material cycle, energy delivery, and technology and innovation management, forming multiple innovation ecological network that are related to energy conservation and emissions reduction industry chain. Since the carbon cap-and-trade system is a mechanism for resource allocation and sustainable development which covers the main areas of CO_2_ emissions in China, it is of great significance to study the impact of China’s national ETS on the ecologicalization of industry. Therefore, this study aims to study the following questions: (1) How to better measure the ecologicalization efficiency of industry in different regions in China under the background of carbon emission reduction? (2) How to explore the effectiveness of ETS in the pilot regions and the impact of ETS on the regional ecologicalization efficiency of industry? (3) How to analyze the acting pattern of the carbon cap-and-trade system on the regional ecologicalization efficiency of industry, and if there are any mediating effects? (4) How to implement ETS to make it work better?

This study focuses on the questions above. First of all, the amount of carbon emissions is considered as undesired outputs, and the regional ecologicalization efficiency of industry before and after the implementation of ETS is measured so as to analyze the effectiveness of ETS, which is beneficial to achieve regional balanced development and implement the low carbon policy more scientifically and effectively. Secondly, exploring how ETS affects regional ecologicalization efficiency of industry could lay a foundation for realizing low-carbon and industrial ecological goals and improving related emission reduction and environmental policies; this study also provides suggestions and strategies for regions with low ecologicalization efficiency of industry to improve efficiency. In general, this study has expanded the research scope of ETS and enriched the evaluation index system of regional ecologicalization efficiency of industry, making the evaluation results of regional ecologicalization efficiency of industry more scientific and reasonable, and providing suggestions for the optimization, adjustment, and sustainable development of China's industrial development under the current low-carbon development background.

The rest of this article is organized as follows: “Literature review” section reviews the research on carbon emissions trading policy and regional ecological efficiency of related literature, the third quarter is introduced in this study; “Research design” section is the method, model building, and data selection; “Analysis of empirical results” section is the description and analysis of the empirical study as well as the robustness test; “Conclusions and policy implications” section is a summary of the full text including some suggestions for policymakers.

## Literature review

### Ecologicalization efficiency of industry and CTS

In 1990, German scholars Schaltegger and Sturm proposed the definition of ecological efficiency for the first time in the academic circle, holding the view that ecological efficiency is the ratio of value increase and environmental change^8^. OECD regards the ecologicalization of industry as the ratio of output (value of products and services) and input (environmental pressure)^9^. Ecologicalization efficiency is the embodiment of ecologicalization of industry which involves the allocation of resources efficiency and the overall operation efficiency, etc. The study of ecologicalization efficiency of industry is of reference value to evaluate the sustainable development of China's industrial economy in balancing economic development, emissions reduction and energy conservation. In many developed countries, ecologicalization of industry has witnessed its social influence on the sustainable development of industrial parks and has guiding significance for the construction of ecological cities^10^. Based on the concept of ecologicalization of industry, Corder et al.^11^ advocated strengthening the waste recycling of mineral resources development in Australia and estimated that the value of metals in end-of-life products is more than AUD6 billion per year. Liu et al.^12^ found that to speed up the ecological transformation of the industrial park, Burnside Industrial Park in Canada not only attached great importance to the reuse, recycling, re-manufacture, leasing, and recycling of industrial materials, such as packaging materials but also produced and sold more and more environmental protection products, which effectively reduced the adverse impact of the industrial park on the environment. The research methods of ecologicalization efficiency of industry mainly include fuzzy Analytic Hierarchy Process (AHP), single ratio method, data envelopment model method, factor analysis method, etc. Fare^13^ used DEA technology to measure environmental technical efficiency, and brought unexpected output into the calculation, laying a foundation for better measuring ecological efficiency. Xiao et al.^14^ used the fuzzy comprehensive evaluation model to comprehensively evaluate the ecological efficiency of enterprises which provided decision-making information for stakeholders and a model which could improve the performance of economy and environment. Under the new normal of China’s economy, Fu^15^ used DEA model to study the ecologicalization efficiency of industry of urban agglomeration, showing the larger the city, the higher the ecological efficiency of urban industry. Jara et al.^16^ proposed a two-step ecological efficiency method combining life cycle assessment and life cycle cost, which provided a new idea for efficiency measurement. Paulo et al.^17^ adopted CE7 to measure the ecological efficiency of oil and gas supply chain enterprises in Brazil, and the results of which show that the ecological efficiency of such enterprises was generally low.

Ecologicalization of industry pays attention to the win–win situation between economy and ecology. However, the industry is prone to the problem of unclear property rights in the process of ecological governance, which leads to the occurrence of external diseconomy. When private cost equals social cost, the problem of external diseconomy can be solved. Therefore, ETS is considered as a tool of environmental regulation, contributing to the study on the acting pattern and principle of ETS on the regional ecologicalization efficiency of industry. Porter hypothesis holds that appropriate environmental regulation is conducive to enterprise innovation, so as to offset environmental protection costs and improve enterprise productivity and profitability^18^. Pigou, a British economist who put forward Pigou tax theory in 1912, believed that polluters should be taxed according to the degree of harm caused by pollution which was later questioned by Coase who believed that a market-oriented mechanism would be more conducive to emission reduction. In 1968, Dales proposed the trading institution of pollution discharge rights, specifying that under the premise of not exceeding allowable pollution loads, enterprises can trade pollution discharge rights as a commodity^19^. The carbon emissions rights which are inspired by pollution discharge rights are taken as a commodity as well and it is a market-based carbon reduction policy tool. Later, carbon emission trading was widely recognized and was gradually improved. According to Coase theorem, the price of carbon emission rights is determined by market supply and demand which helps to solve external economic problems. When enterprises have to pay for external costs caused by their emissions and realize the internalization of external costs, the pareto optimization of the society can be achieved.

In 2005, the world's first EU ETS was established, which adopted a decentralized governance model and greatly reduced global greenhouse gas emissions. In contrast, China's implementation of ETS was later, and there were few studies on carbon emission trading. Yang et al.^20^ constructed a two-stage game model and found that the policy of carbon emission rights trading cooperation and allowable emission permits could be beneficial to reduce greenhouse gas emissions. Sun et al.^21^ evaluated the carbon emission trading system of Beijing, Shanghai, Tianjin, and Shenzhen by combining the fuzzy evaluation method with Delphi method, finding that the operating effect of Shanghai’s carbon emission trading was the best. Zhang et al.^22^ using system dynamics, concluded that carbon trading mechanism could be beneficial to the carbon emission reduction process, while it could have bad effects on economy as well. Zhang et al.^23^ used DID model, stochastic frontier analysis, Distance function, and so on to explore the carbon mitigation effect and potential cost savings of carbon emission trading, believing that energy efficiency had a greater impact on the carbon mitigation effect. Lv et al.^24^ use the event research method and benchmark model to evaluate the effect of ETS from the perspective of enterprise innovation, reviewing that high carbon trading price could promote the improvement of enterprise innovation technology.

In addition to the carbon trading plan, scholars have also studied other government means or perspectives to promote the sustainable development of the industry, such as environmental regulation and green premium. Zhang et al.^25^ used a panel data model to calculate the impact of three environmental regulations that are connected with the administration, market, and public participation on industrial green growth index. The results show that environmental regulations that are connected with administration and market play a significant role in industrial green growth. To meet the challenge of ecologicalization of industry in Spain, Calvo et al.^26^ designed a management model to analyze the impact of incentive policies on the use of C & D waste by management departments and believed that the comprehensive use of supervision, environmental tax, and other general measures is more effective. Zhu et al.^27^ studied emission reduction methods taking green premium into account, including End-of-Pipe Technology and Clean Technology. Finally, it is suggested that the government regulate the trading price of emission rights by investigating the emission reduction costs of various industries, so as to urge emission-dependent producers to reduce emission initiatively^27^. To reduce the ecological environment loss caused by industrial development, Cai et al.^28^ established the ecological compensation standard at the government level after the energy loss estimation of industrial solid waste and verified that the compensation standard of phosphogypsum was 37.4 yuan/ton. Belaud et al.^29^ developed a technical method toolbox jointly facilitated by public laboratories, urban planning companies, and the French government environment agency to promote the implementation of ecologicalization of industry in French industrial parks.When exploring the issue of power supply guarantee, Li^30^ emphasized the correct understanding of green premium in energy transition, and he believed that mechanism innovation reform was very important.

### Review of research status

Domestic and foreign researchers have achieved certain results in the research of ecologicalization of industry and low-carbon policies. At present, the assessment of ETS is more concentrated on the analysis of policy itself and related case analysis, putting forward the problems facing our country under the current policy environment and mechanism analysis. Although some scholars measure the reduction effect of policy through empirical analysis, their methods are limited and lack in-depth discussion. In contrast, foreign scholars have refined and decomposed the research content, focusing more on the discussion of the concrete implementation of the policy itself. The research on industrial ecologilcalization mostly focuses on the construction of the conceptual framework and evaluation principles and the establishment of incentive means. Besides, most of them are case studies and few scholars study the efficiency of industrial ecologilcalization at the regional level. It lacks further discussion on the influencing factors and internal mechanism of the efficiency of regional industrial ecologilcalization. Compared with foreign researchers, domestic researchers are late in the study on carbon emission trading policy and ecologicalization efficiency of industry, and there are few systematic application research. Few researchers measure the impact of carbon emission trading policy on the regional industrial ecologilcalization efficiency. Given the shortcomings of existing studies, this study reviews the concept of regional ecologicalization efficiency of industry, explores the effectiveness of ETS in pilot areas from a new perspective, and further studies the impact of ETS on the regional ecologicalization efficiency of industry.

## Research design

### Model establishing

This study uses the dual difference model to evaluate the impact of the implementation of ETS on the regional ecologicalization efficiency of industry. The implementation of ETS is taken as a quasi-natural experiment, and 2013 is the time node when the pilot carbon emission trading policy is officially implemented. The effectiveness of ETS is observed by comparing the development of ecologicalization efficiency of industry in pilot and non-pilot areas before and after the implementation of ETS. The setting model is shown in Eq. (1):

1 $$ {\text{IEE}}_{{{\text{it}}}} = \beta_{0} + \beta_{1} {\text{C}}_{i} + \beta_{2} Y_{{\text{i}}} + \beta_{3} \left( {{\text{R}}_{{\text{i}}} *{\text{Y}}_{{\text{i}}} } \right) + \beta_{4} {\text{K}}_{{{\text{it}}}} + \alpha_{{\text{i}}} + \lambda_{{\text{i}}} + \varepsilon_{{{\text{it}}}} $$

In Eq. (1), IEE_it_ represents regional ecologicalization efficiency of industry, i represents the area, t indicates time, R_i_ is the zone virtual variable, Y_i_ represents the year virtual variables, β_3_ is a dual differential estimate, K_it_ represents control variables including seven factors: the level of economic development, technological progress, energy consumption structure, state-owned, opening up to the level of economic development, industrial structure, and population density. α_i_ represents time fixed effects, λ_i_ represents time-fixed effect, ε_it_ represents random interference.

### Variable selection and data description

#### Variable selection

Explained variable: regional ecologicalization efficiency of industry is a comprehensive index reflecting the coordinated development of the regional environment and economy, which emphasizes the realization of economic output maximization on the basis of minimizing environmental pollution and maximizing resource utilization. Considering that the ordinary DEA model cannot include the unexpected outputs, and many decision units may be effective, leading to the failure of sorting, and the ordinary SBM model may have the problem that the efficiency value of multiple decision units is 1, this study selects the super-SBM model with variable returns to scale to measure the regional ecologicalization efficiency of industry. This model can not only avoid the above problems but also reflect the actual situation of ecologicalization efficiency of industry in each region. The model form is shown in Eq. (2).

2 $$ \begin{gathered} \min \rho = \frac{{1 + \frac{1}{m}\sum\limits_{i = 1}^{m} {{\raise0.7ex\hbox{${si^{ - } }$} \!\mathord{\left/ {\vphantom {{si^{ - } } {xx_{ik}}}}\right.\kern-\nulldelimiterspace} \!\lower0.7ex\hbox{${x_{ik}}$}}} }}{{1 - \frac{1}{s}\sum\limits_{i = 1}^{m} {{\raise0.7ex\hbox{${sr^{ + } }$} \!\mathord{\left/ {\vphantom {{sr^{ + } } {yx_{rk}}}}\right.\kern-\nulldelimiterspace} \!\lower0.7ex\hbox{${yx_{rk}}$}}} }} \hfill \\ s.t.\sum\limits_{j = 1,j \ne k}^{n} {x_{ij}\lambda_{j} - s_{i}^{ - } \le x_{ik}} \hfill \\ \sum\limits_{j = 1,j \ne k}^{n} {y_{ij}\lambda_{j} + s_{r}^{ + } \le yx_{rk}} \hfill \\ \sum\limits_{j = 1,j \ne k}^{n} {\lambda _{j}} = 1 \hfill \\ \lambda ,s_{r}^{ + } ,s_{i}^{ - } \ge 0 \hfill \\ i = 1,2,...,m;r = 1,2,...,q;j = 1,2,...,n(j \ne k) \hfill \\ \hfill \\ \end{gathered} $$

Assuming a total of N decision units, each decision unit has m inputs and q outputs, x represents the input variable, y indicates the output variable, the k_th_ is expressed as x_ik_, y_rk_. s_r_^+^ and s_i_^-^ represent the relaxation variable of input–output. The specific input–output indicators are shown in Table 1, among which the calculation of carbon emissions is based on the energy consumption of fossil fuels and the corresponding carbon emission coefficient of each province following the “2006 IPCC Guidelines for National Greenhouse Gas Inventories”.

**Table 1:** Evaluation index system of regional ecologicalization efficiency of industry.

Categories	Name	Definition	Unit
Input	Quantity of employment	Reflects the total available labor force	Thousands of people
	Fixed investments	Reflects capital input	Ten thousand yuan
	Total energy consumption	Reflects the energy input needed for industrial development	Ten thousand tons of standard coal
	Construction land area	Reflective land input	km^2^
	Total water content	Reflect industrial water resources use	Hundred million cubic meters
de Desirable output	Industrial increment	Reflects the level of economic output of an industry	100 million yuan
Undesirable output	Carbon emission	Reflecting greenhouse gas emissions	10,000 tons
	Solid waste production	Reflects the discharge of solid waste	10,000 tons
	discharge amount of wastewater	Reflect the discharge of industrial wastewater	Ten thousand tons of standard coal
	Emission quantity of so_2_	Reflect the emission of SO_2_ in industry	Ton

Core explanatory variable: the core explanatory variable is a dummy variable: R_i_*Y_i_. Only when Y is greater than or equal to 2013 and it is located in a carbon emission trading pilot region, namely, a low-carbon pilot region, R_i_*Y_i_ is 1; otherwise, R_i_*Y_i_ is 0. The coefficient of the cross-product term is a dual difference estimator, which can reflect the effectiveness of ETS on the regional ecologicalization efficiency of industry.

Control variable: to rule out other factors which may influence the results, this study selects the 7 control variables that influence the regional ecologicalization efficiency of industry: (1) economic development level (GDP): the mutual transformation between economic development and ecological environment protection is aimed to achieve the good life of people. This study measures GDP by gross regional industry, that is, the sum of the added value of the industry. (2) Technological progress (tps): technological progress can promote regional technological reform and innovation, improve production efficiency and factors of production, and expand economies of scale. In this study, technological market turnover is used to measure, specifically refer to the actual total amount of technological transactions. (3) Energy consumption structure (ecs) is an important indicator of regional economic development and sustainable development, which is closely related to the regional ecologicalization of industry. In this paper, the proportion of coal consumption in energy consumption is used to measure it. (4) State-owned economic development (sed): state-owned economic development plays an important role in guiding industrial-technological progress, improving the level of ecologicalization of industry, and promoting the development of the modern industrialized economy. In this study, the proportion of state-owned economic income in the total GDP is used to measure it. (5) Opening to the outside world (oul) helps to strengthen the flow and integration of innovative elements in the international, the construction of opening to the outside world system is particularly important for the healthy and sustainable development of China's economy, this study is measured by the proportion of foreign investment in the total GDP, specifically refers to the total investment of foreign investors in China. (6) Industrial structure level (isl): the gap in the process of industrialization leads to obvious differences in the level of industrial structure among different regions in China, which is multi-tiered. In this study, the proportion of the added value of the secondary industry in the total value is used to measure it. (7) Population density (pd), which can reflect the density of regional population, is closely related to the level of economic development and the quality of urbanization. It is measured by the number of people per unit of land area in a region, specifically the number of people living per square kilometer of land. As shown in Table 2.

**Table 2:** Description of control variables.

Control variables	Description
Economic development level (GDP)	Gross regional industry (100 million yuan)
Technological progress (tps)	Technological market turnover (10,000 yuan)
Energy consumption structure (ecs)	The proportion of coal consumption in energy consumption (%)
State-owned economic development (sed)	The proportion of state-owned economic income in the total GDP (%)
Opening to the outside world (oul)	The proportion of foreign investment in the total GDP (%)
Industrial structure level (isl)	The proportion of the added value of the secondary industry in the total value (%)
Population density (pd)	The number of people living per square kilometer of land (square kilometers)

#### Source of data

Due to incomplete data i of Tibet, panel data of 30 provinces and cities in China from 2007 to 2019 were selected for the study. The data was collected from statistical yearbooks of different provinces and cities, “China environmental statistical yearbook”, “China statistical yearbook”, “China energy statistical yearbook”, “China state-owned assets supervision and management yearbook”, etc. The descriptive statistical results of variables are shown in Table 3. IEE stands for ecologicalization efficiency of industry, Other abbreviations are explained above.

**Table 3:** Descriptive statistics of variables.

Variable	Observed value	All samples		Treatment group		Control group	
		Mean	St.dev	Mean	St.dev	Mean	St.dev
IEE	390	0.511	0.328	0.962	0.343	0.399	0.204
GDP	390	20,576.856	18,122.628	26,477.421	21,380.977	19,101.715	16,932.270
tps	390	2,788,077.506	6,320,426.802	8,613,818.270	11,727,477.700	1,331,642.315	2,296,731.473
ecs	390	0.948	0.414	0.573	0.202	1.042	0.400
sed	390	0.342	0.271	0.488	0.324	0.305	0.248
oul	390	0.466	0.611	0.852	0.543	0.369	0.589
isl	390	46.311	21.197	41.209	10.801	47.587	22.911
pd	390	2816.326	1198.358	2547.103	926.154	2883.631	1249.455

## Analysis of empirical results

### Benchmark regression results

#### Parallel trend test

The premise of using DID is that the treatment group and control group meet the assumptions of parallel trend, which means that before ETS is officially implemented, the evolution trend of ecologicalization efficiency of industry of the control group and the experimental group is consistent and does not show a systematic difference. This study uses a more rigorous empirical test in parallel trend test: if the interaction coefficient is not significant and is different from zero before the implementation of ETS; and if the interaction coefficient is significant and is different from zero after the implementation of ETS, it indicates that there is no significant difference in ecologicalization efficiency of industry between the control group and the experimental group before the implementation of ETS. Results are shown in Table 4: before ETS was officially implemented, the difference coefficient was not significant; after the official implementation of ETS in 2013, the difference coefficient was significant and not equal to 0, and the ecologicalization efficiency of industry was improved significantly, which met the parallel trend of the DID. Therefore, it is scientific and reasonable to evaluate the effectiveness of ETS with DID.

**Table 4:** Parallel trend test.

Y	Coef	Control variable	Fixed effect	N
pre_6	0.015711 (0.86)	Yes	yes	390
pre_5	− 0.0060199 (0.946)	Yes	Yes	390
pre_4	0.0093869 (0.916)	Yes	Yes	390
pre_3	0.0361118 (0.685)	Yes	Yes	390
pre_2	− 0.0343934 (0.699)	Yes	Yes	390
time_1	0.0959477 (0.281)	Yes	Yes	390
time_2	0.1176363 (0.186)	Yes	Yes	390
time_3	0.2500371*** (0.005)	Yes	Yes	390
time_4	0.2693522*** (0.003)	Yes	Yes	390
time_5	0.254908*** (0.004)	Yes	Yes	390
time_6	0.3745259*** (0.000)	Yes	Yes	390

***, ** and * represent significance levels of 1%, 5% and 10% respectively, with P values in brackets.

#### Dynamic effect analysis

**Figure 1:**
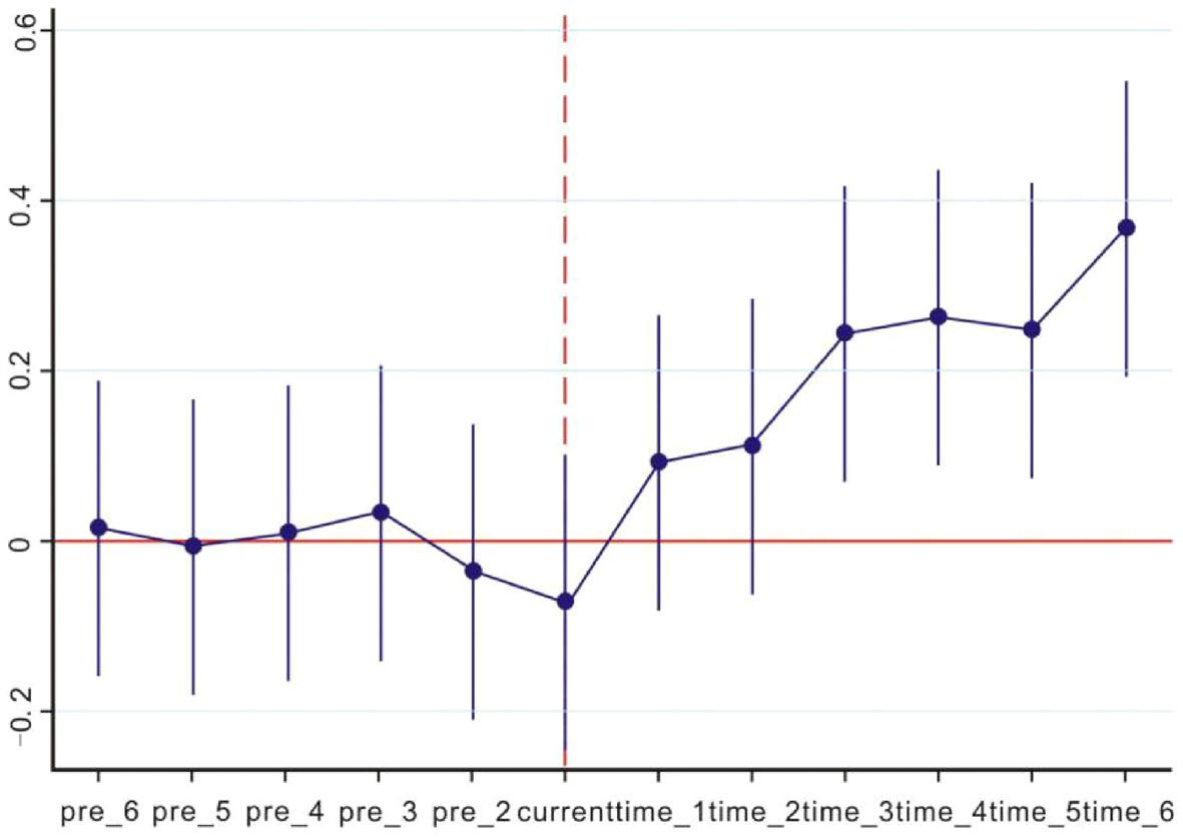
Dynamic analysis diagram.

To compare the conditions of the experimental group and the control group before and after the implementation of ETS, dynamic graphs are drawn in this study, as shown in Fig. 1, which shows the impact of ETS on the regional ecologicalization efficiency of industry. The vertical line represents a 95% confidence interval and the broken line shows the marginal effect of regional ecologicalization efficiency, which means that the confidence interval contains is 0 before ETS’s implementation, and the result is not significant. In contrast, after 2013, the effect of ETS became apparent, the marginal effect gradually increased and the results became significant, perhaps owing to the implementation of ETS.

### The effect of ETS on ecologicalization efficiency of industry

Controlling time effect and fixed effect, this study collected the data of pilot and non-pilot provinces of ETS from 2007 to 2019 to analyze the impact of ETS on the regional ecologicalization efficiency of industry and regional heterogeneity. The results are shown in Table 5. According to the results in the first column, ETS has significantly promoted the regional ecologicalization efficiency of industry, and the national implementation of ETS has achieved remarkable results. Compared with the regions that are not ETS pilot areas, the ecologicalization efficiency of industry of pilot provinces and cities has increased by 35%. Results also show that ETS has different effects on the ecologicalization efficiency of industry in different regions. Specifically, ETS significantly promoted regional ecologicalization efficiency of industry in the eastern and central regions, and the efficiency in the eastern region was more significant than that of the central region. However, the impact of ETS on the regional ecologicalization efficiency of industry in the western region was negative which may result from the fact that compared to the central and western regions, the east region has better economic development, advanced technology, and lots of talents that can respond to the implementation of ETS, accelerate the upgrade of industries, and improve the utilization level of regional resources. There are many traditional industries in the central and western regions, and the development of scientific and technological levels as well as the resource utilization efficiency there are relatively slow. Besides, it is difficult for the central and western regions to adapt to ETS in a short-term of time leading to the failure of improving the regional ecologicalization efficiency of industry in a short time.

**Table 5:** Influence of ETS on ecologicalization efficiency of industry.

Variable	All region	East	Midland	West
Ri*Yi	0.354*** (0.043)	0.351*** (0.058)	0.061* (0.037)	− 0.003 (0.013)
GDP	0.161** (0.066)	0.124** (0.089)	0.523 (0.316)	0.134 (0.195)
tps	− 0.008* (0.005)	− 0.255* (0.155)	− 0.064* (0.027)	0.461* (0.279)
ecs	0.001 (0.003)	0.002 (0.002)	− 0.028* (0.016)	0.014* (0.005)
sed	− 0.003 (0.002)	− 0.002 (0.009)	0.009* (0.004)	0.005* (0.002)
oul	0.004* (0.001)	0.003* (0.002)	0.003 (0.005)	0.004 (0.007)
isl	0.008*** (0.003)	0.001** (0.001)	0.001*** (0.000)	0.008 (0.082)
pd	− 0.168*** (0.062)	− 0.279 (0.196)	0.018 (0.088)	− 0.056 (0.208)
Control variable	Yes	Yes	Yes	Yes
Individual fixed Effect	Yes	Yes	Yes	Yes
Time fixed effect	Yes	Yes	Yes	Yes
N	390	143	104	143
R-squared	0.232	0.125	0.407	0.338

***, ** and * represent significance levels of 1%, 5% and 10% respectively, standard error is in brackets.

### Robustness test

#### Propensity matching score—double difference method (PSM-DID)

The assumption of homogeneity and randomness between the control group and the experimental group is the premise of using the DID model. However, due to the large economic and regional differences among provinces and cities, there may be systematic differences between the experimental group and the control group, which may cause deviations in the results. Therefore, the data after propensity score matching is used in this study, making the matched individuals have no other significant differences unless they have been treated or not. The dual difference is conducted again to avoid self-selection bias, and the robustness of the above results is verified according to the measurement results. Control variables were used to match characteristic variables, the experimental group was matched with the control group, and the Logit model was adopted to delete the samples that fail to meet the matching criteria. After the matching, there are 168 observation values. The regression results of PSM-DID model show that, ETS has positive effects on the regional ecologicalization of industry (0.460***), which again proves that the conclusion that ETS improves regional ecologicalization of industry efficiency is reliable. The results are shown in Table 6.

**Table 6:** The result of the PSM-DID.

Variable	All region	East	Midland	West
Ri*Yi	0.460*** (0.132)	0.297*** (0.044)	0.084** (0.042)	− 0.010 (0.009)
GDP	0.205** (0.113)	0.243 (0.104)	0.471** (0.237)	0.099 (0.337)
tps	− 0.015* (0.009)	− 0.302* (0.183)	− 0.044 (0.009)	0.517* (0.313)
ecs	0.052* (0.031)	0.028 (0.022)	− 0.106 (0.104)	0.041* (0.023)
sed	− 0.017 (0.114)	− 0.011 (0.009)	0.041*** (0.004)	0.007* (0.004)
oul	0.102* (0.054)	0.092 (0.310)	0.088* (0.050)	0.039* (0.021)
isl	0.015** (0.008)	0.104* (0.047)	0.161** (0.081)	0.124 (0.148)
pd	− 0.206** (0.010)	− 0.331* (0.246)	0.008 (0.103)	− 0.105 (0.662)
Control variable	Yes	Yes	Yes	Yes
Individual fixed effect	Yes	Yes	Yes	Yes
Time fixed effect	Yes	Yes	Yes	Yes
N	168	51	69	48
R-squared	0.304	0.227	0.316	0.402

#### Counterfactual test

To verify the robustness of the results again, six provinces and cities are randomly selected as experimental groups for multiple tests to construct new dummy variables of ETS, and the DID model was used again to verify the credibility of the above results. Four random samples were conducted in this study, and the results are shown in Table 7. It can be seen that the results are not significant, which also reversely proves that ETS improves the regional ecologicalization efficiency of industry.

**Table 7:** Counterfactual test results.

Variable	Counterfactual test			
	(1)	(2)	(3)	(4)
Did	0.412651 (0.268)	0.3124455 (0.543)	0.5132819 (0.443)	0.2924251 (0.472)
Control variable	Yes	Yes	Yes	Yes
Individual fixed effect	Yes	Yes	Yes	Yes
Time fixed effect	Yes	Yes	Yes	Yes
N	390	390	390	390
R-squared	0.3255	0.0927	0.4611	0.25632

### Acting pattern analysis of ETS on the regional ecologicalization efficiency of industry

First, ETS may improve the regional ecologicalization efficiency of industry through industrial structure optimization and upgrading. Promoting upgrading of the industrial structure is one of the important approaches of social and economic development during the 14th Five-Year Plan formulation and is the only way to promote low-carbon and sustainable development of modern national industries. The upgrading of the industrial structure has been promoted to the national strategic level, contributing to the healthy development of the national economy system. ETS bring costs and benefits to enterprises, forcing them to transform and upgrade, increase investment in environmental protection and use clean energy, and accelerate the pace of energy conservation and emission reduction^31^. Second, ETS may improve the regional ecologicalization efficiency of industry through the coordinated agglomeration of resources. Marshall's theory of scale economy, Krugman's theory of new economic geography, Weber's theory of agglomeration economy, Coase's transaction cost theory, and so on reflect the importance of resource aggregation of economic activities through cost-saving, resource sharing, and other ways to improve industrial input–output efficiency, enhance industrial competitiveness, increase regional comprehensive strength and strengthen the competitive advantage of regional industrial clusters^32^. The benefits generated by resource aggregation far exceed the sum of benefits generated by various industries in the decentralized state. Under the pressure of ETS, enterprises may alleviate the mismatch between labor and capital through the collaborative aggregation of industrial resources, aiming to improve economic benefits and regional resource allocation efficiency and promote regional ecologicalization efficiency of industry. Third, ETS may improve the regional ecologicalization efficiency of industry by supporting ecological optimization. The sustainable development of the ecological environment is closely related to emission reduction policy. To alleviate the bad effects on the ecology, environmental protection is more and more brought to the attention of society and government. Policies for ecological protection have been introduced to reduce pollution^20^. All regions take effective and targeted measures to control environmental pollution and optimize the investment structure in light of their actual conditions. The purpose of ecological optimization is to improve the regional environment and strengthen pollution control which is one of the important parts of China's fiscal spending. The government must guide the market to carry out ecological protection and environmental governance according to ETS. Studies have found that a low-carbon pilot policy helps to enhance the level of regional pollution control, promote the harmonious development of regional economy and environment, and then improve the regional ecologicalization efficiency of industry.

To explore the transmission mechanism of ETS on the regional ecologicalization of industry efficiency, Baron and Kenny (1986)'s mediating effect model was referred to explore and verify whether there exists a structural optimization upgrade effect, resource synergistic agglomeration effect, ecological optimization support effect when ETC promotes regional ecologicalization efficiency of industry. Table 8 shows the regression results of the influence mechanism of ETS on the regional ecologicalization efficiency of industry. This study refers to the definition and research of industrial optimization and upgrading by Wang Qunwei, Huang Xianglan, and others, and the proportion of tertiary industry added value accounting for industrial added value is selected to measure the effectiveness of industrial optimization and upgrading. For resource synergistic agglomeration effect, this study refers to the calculation methods of Cui Shuhui, Chen Jianjun et al. and adopts the collaborative aggregation index of manufacturing and producer services to measure the collaborative aggregation effect of resources, which effectively avoids the scale difference between different regions. It can be seen from the table that the implementation of ETS has significantly influenced the three effects proposed by this study: the optimization and upgrading effect of industrial structure, the synergistic aggregation effect of resources, and the support effect of ecological optimization. In addition, ETS has a positive and significant impact on the regional ecologicalization efficiency of industry. The results in Columns 3, 5, and 7 of the table show the industrial optimization and upgrading effect, resource synergistic aggregation effect, structural upgrading effect, and resource allocation effect generated in the process of low-carbon pilot policy operation can significantly promote regional ecologicalization efficiency of industry and have an obvious intermediary effect. The mediating effect produced by industrial structure optimization and upgrading is about 0.042, the mediating effect produced by resource synergy agglomeration is about 0.148, and the mediating effect produced by ecological optimization support is about 0.166. According to the Sobal test results, all of them have passed the test, indicating that the above results are reliable.

**Table 8:** Mediating effect test results.

Variable	M1		M2		M3		
	IEE	isu	IEE	rca	IEE	eos	IEE
Treat × D	0.354*** (0.043)	0.414** (0.213)	0.312*** (0.125)	0.348*** (0.095)	0.206*** (0.072)	0.284*** (0.103)	0.188 *** (0.044)
isu			0.101*** (0.029)				
rca					0.425*** (0.112)		
eos							0.585*** (0.074)
_cons	0.372*** (0.079)	0.965*** (0.088)	0.416*** (0.053)	1.775*** (0.063)	0.103*** (0.042)	0.983 *** (0.031)	0.337*** (0.046)
Control variable	Yes	Yes	Yes	Yes	Yes	Yes	Yes
Individual fixed effect	Yes	Yes	Yes	Yes	Yes	Yes	Yes
Time fixed effect	Yes	Yes	Yes	Yes	Yes	Yes	Yes
N	390	390	390	390	390	390	390
R-squared	0.326	0.108	0.410	0.204	0.711	0.068	0.394
Sobel		0.064***		0.203***		0.043***	
Goodman-1		0.064***		0.203***		0.043***	
Goodman-2		0.064***		0.203***		0.043***	

***, ** and * represent significance levels of 1%, 5% and 10% respectively, standard error is in brackets.

## Conclusions and policy implications

This article is based on the panel data of 30 provinces and cities in China from 2007 to 2019. First, the SBM-DEA model is used to measure the ecological efficiency of regional industries in various provinces and cities. Then, DID is used to conduct a quasi-natural experiment.

At the same time, PSM-DID model is used to test the influence of ETS on the regional ecologicalization efficiency of industry, and three hypotheses of effects are proposed to explore the acting pattern of ETS on the regional ecologicalization efficiency of industry. The results show that: (1) there is a significant positive correlation between the implementation of ETS and regional ecologicalization efficiency of industry, showing that ETS is effective and plays a positive role in the improvement of regional ecologicalization efficiency of industry. (2) The implementation of carbon emission trading policy has a certain regional heterogeneity, and the improvement of ecologicalization efficiency of industry is more obvious in the eastern region, followed by the central region, and the effect is not obvious in the western region. (3) Through mechanism analysis, it can be concluded that ETS can improve regional ecologicalization efficiency of industry by promoting the high-level of regional industrial structure, improving the level of industrial resource synergy and environmental condition. Based on the above research conclusions, this study puts forward the following suggestions for policymakers:

The development of the national carbon market needs the joint efforts of all provinces and cities. All regions should pay more attention to industrial innovation and upgrading, take ecologicalization of industry as the guidance, adjust the economic structure, eliminate backward production capacity, transform traditional industries, convert old and new kinetic energy, promote the development of low-carbon industry and tertiary industry, reasonably arrange the secondary industry, promote industrial low-carbon transformation, and vigorously promote the development of green emerging industries. For the central and western regions with a poor foundation, it is necessary to strengthen cooperation and exchanges with developed regions. The government can also give appropriate resource support to stimulate their emission reduction potential. For the eastern region with a good foundation, it is important to play a leading role and use advantageous resources to carry out multi-level, multi-channel, and multi-field technology cooperation, so as to further improve the utilization rate of resources. All regions need to formulate environmental policies and mechanisms that adapt to the regional development structure and resource endowment and implement differentiated emission reduction mechanisms according to the characteristics of enterprises and ownership structure. All innovation subjects should lead the process of low-carbon scientific and technological innovation, and upgrade and transform low-carbon innovation achievements and technologies. According to market demand, all subjects upgrade low-carbon innovation and technologies, and shift from "made in China" to "created in China". As participants, contributors, and leaders in the construction of ecological civilization, all subjects promote the ecologicalization efficiency of industry in various regions of China and contribute to global sustainable development. What’s more, all regions should speed up the process of industrial integration and enhance the degree of industrial agglomeration, especially promote the collaborative agglomeration of manufacturing and producer services, and form a dynamic, open, comprehensive, stable, and coordinated urban agglomeration and industrial chain to realize the sustainable development of ecological economy and promoting the construction of carbon market. The actual practice can reduce factor costs and information costs improve production efficiency for enterprises, and expand the manufacturing service industrial chain. Besides, the sharing of resource allocation, human capital, scientific research, and innovation will help enterprises make more application achievements. At present, only the power industry has participated in the national carbon trading market so it is necessary to expand the scope of the industry, reduce the threshold of carbon trading and encourage non-state-owned and state-owned enterprises to participate in the market, and expand the main body of market trading so as to improve the vitality of the market. The state should coordinate with governments at all levels, strictly control the total amount of carbon emissions and conduct regular verification and supervision according to environmental development and market economic situation. To achieve effective emission reduction, the government should formulate laws and reasonable carbon trading plans, improve carbon-emission trading rights allocation rules and carbon pricing mechanism, regulate the market in a further way, and lay a foundation for the good operation of the carbon trading market.

## Data Availability

Data derived from public domain resources: the data that support the findings of this study are available in [China environmental statistical yearbook] at [http://www.stats.gov.cn/ztjc/ztsj/hjtjzl/], [China statistical yearbook] at [http://www.stats.gov.cn/tjsj/ndsj/], [China energy statistical yearbook] at [http://cwres.ncu.edu.cn/s/net/cnki/data/G.https/yearbook/Single/N2021050066], [China population and employment statistics yearbook] at [https://www.yearbookchina.com/navibooklist-n3022013208-1.html].
